# Structural and electrical characteristics of high-*κ* Er_2_O_3_ and Er_2_TiO_5_ gate dielectrics for a-IGZO thin-film transistors

**DOI:** 10.1186/1556-276X-8-18

**Published:** 2013-01-08

**Authors:** Fa-Hsyang Chen, Jim-Long Her, Yu-Hsuan Shao, Yasuhiro H Matsuda, Tung-Ming Pan

**Affiliations:** 1 Department of Electronics Engineering, Chang Gung University, 333, Taoyuan, Taiwan; 2 Division of Natural Science, Center for General Education, Chang Gung University, 333, Taoyuan, Taiwan; 3 Institute for Solid State Physics, University of Tokyo, 277–8581, Chiba, Japan

**Keywords:** Amorphous InGaZnO, Thin-film transistor, Er_2_O_3_, Er_2_TiO_5_

## Abstract

In this letter, we investigated the structural and electrical characteristics of high-*κ* Er_2_O_3_ and Er_2_TiO_5_ gate dielectrics on the amorphous indium-gallium-zinc-oxide (a-IGZO) thin-film transistor (TFT) devices. Compared with the Er_2_O_3_ dielectric, the a-IGZO TFT device incorporating an Er_2_TiO_5_ gate dielectric exhibited a low threshold voltage of 0.39 V, a high field-effect mobility of 8.8 cm^2^/Vs, a small subthreshold swing of 143 mV/decade, and a high *I*_on_/*I*_off_ current ratio of 4.23 × 10^7^, presumably because of the reduction in the oxygen vacancies and the formation of the smooth surface roughness as a result of the incorporation of Ti into the Er_2_TiO_5_ film. Furthermore, the reliability of voltage stress can be improved using an Er_2_TiO_5_ gate dielectric.

## Background

Amorphous indium-gallium-zinc-oxide (a-IGZO) thin-film transistors (TFTs) are being extensively explored as a replacement for amorphous and polycrystalline silicon TFTs in large-area display technologies, such as active-matrix liquid crystal display devices and active-matrix organic light-emitting displays [1]. This is due to their high field-effect mobility, low leakage current, excellent optoelectronic characteristics, good uniformity and stability, and low temperature fabrication [2].

To achieve a high drive current at a low gate voltage, we can either employ high-*κ* materials or thinner gate dielectrics [3]. However, the decrease in the thickness of gate dielectric is limited due to the occurrence of electron tunneling. Consequently, high-*κ* gate dielectric materials, including Al_2_O_3_[4], ZrO_2_[3], Y_2_O_3_[5], and HfO_2_[6], have been studied to reduce the electron tunneling and maintain the large capacitance. However, HfO_2_ dielectric film has a critical disadvantage of high charge trap density between the gate electrode and gate dielectric, as well as the gate dielectric and channel layer [7]. Recently, rare earth (RE) oxide films have been extensively investigated due to their probable thermal, physical, and electrical performances [6]. To date, the application of RE oxide materials as gate dielectrics in a-IGZO TFTs has not been reported. Among the RE oxide films, an erbium oxide (Er_2_O_3_) film can be considered as a gate oxide because of its large dielectric constant (approximately 14), wide bandgap energy (>5 eV), and high transparency in the visible range [8,9]. The main problem when using RE films is moisture absorption, which degrades their permittivity due to the formation of low-permittivity hydroxides [10]. The moisture absorption of RE oxide films may be attributed to the oxygen vacancies in the films [11]. To solve this problem, the addition of Ti or TiO_*x*_ (*κ* = 50 to approximately 110) into the RE dielectric films can result in improved physical and electrical properties [12]. In this study, we compared the structural and electrical properties of Er_2_O_3_ and Er_2_TiO_5_ gate dielectrics on the a-IGZO TFT devices.

## Methods

The Er_2_O_3_ and Er_2_TiO_5_ a-IGZO TFT devices were fabricated on the insulated SiO_2_/Si substrate. A 50-nm TaN film was deposited on the SiO_2_ as a bottom gate through a reactive sputtering system. Next, an approximately 45-nm Er_2_O_3_ was deposited by sputtering from an Er target, while an Er_2_TiO_5_ thin film (approximately 45 nm) was deposited through cosputtering using both Er and Ti targets at room temperature. Then, postdeposition annealing was performed using furnace in O_2_ ambient for 10 min at 400°C. The a-IGZO channel material (approximately 20 nm) was deposited at room temperature by sputtering from a ceramic IGZO target (In_2_O_3_/Ga_2_O_3_/ZnO = 1:1:1). Top Al (50 nm) source/drain electrodes were formed by a thermal evaporation system. The channel width/length of examined device was 1,000/200 μm. The film structure and composition of the dielectric films were analyzed using X-ray diffraction (XRD) and X-ray photoelectron spectroscopy (XPS), respectively. The surface morphology of the films was investigated by atomic force microscopy (AFM). The capacitance-voltage (*C*-*V)* curves of the Al/Er_2_O_3_/TaN and Al/Er_2_TiO_5_/TaN devices were measured using a HP4284 LCR meter. The electrical characteristics of the a-IGZO TFT device were performed at room temperature using a semiconductor parameter Hewlett-Packard (HP) 4156C (Palo Alto, CA, USA). The threshold voltage (*V*_TH_) was determined by linearly fitting the square root of the drain current versus the gate voltage curve. Field-effect mobility (*μ*_FE_) is derived from the maximum transconductance.

## Results and discussion

**Figure 1:**
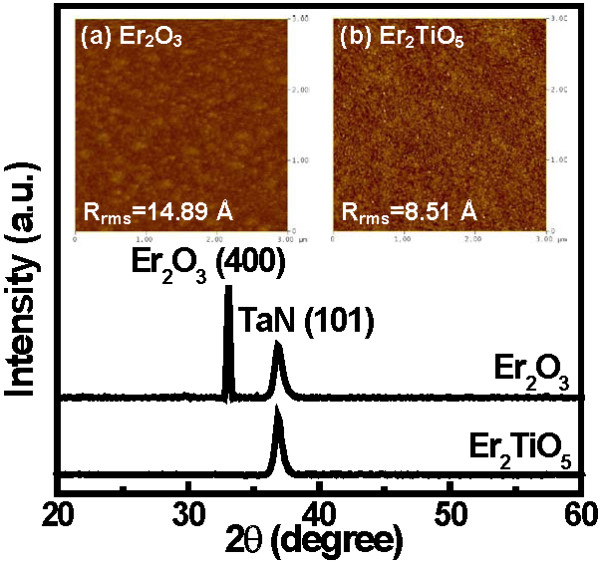
**XRD patterns of Er**_**2**_**O**_**3 **_**and Er**_**2**_**TiO**_**5 **_**dielectric films.** Insets show AFM surface images of (**a**) Er_2_O_3_ and (**b**) Er_2_TiO_5_ films.

Figure 1 displays the XRD patterns of the Er_2_O_3_ and Er_2_TiO_5_ thin films deposited on the TaN/SiO_2_/Si substrate. A strong Er_2_O_3_ (400) and weak TaN (101) peaks appeared in the Er_2_O_3_ film, while only TaN (101) reflection peak was presented in the Er_2_TiO_5_ film, revealing that Er_2_TiO_5_ thin film was amorphous. The insets (a) and (b) of Figure 1 depict the AFM images of the Er_2_O_3_ and Er_2_TiO_5_ thin films, respectively. The Er_2_O_3_ sample shows a higher surface roughness compared with the Er_2_TiO_5_ sample. This is attributed to the increase in the growth of the grain size, which is consistent with the XRD result. Another cause for a rough surface is the nonuniform volume expansion of Er_2_O_3_ film because of the nonuniform moisture absorption of the film [10].

**Figure 2:**
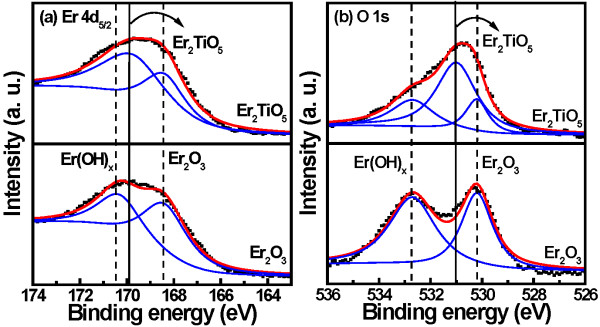
**XPS spectra of (a) Er 4*d***_**5/2 **_**and (b) O 1*s *for Er**_**2**_**O**_**3 **_**and Er**_**2**_**TiO**_**5 **_**dielectric films.**

Figure 2a,b presents the Er 4*d*_5/2_ and O 1*s* XPS spectra of the Er_2_O_3_ and Er_2_TiO_5_ dielectric films, respectively. In the three sets of spectra, each fitting peak is assumed to follow the general shape of the Lorentzian-Gaussian function: one peak represents the Er-OH bonds (located at 170.4 eV), the second the Er-O-Ti bonds (located at 169.9 eV), and the third the Er-O bonds (located at 168.4 eV) [13]. The Er 4*d*_5/2_ peak of the Er_2_O_3_ film has two intensity peaks corresponding to Er_2_O_3_ and Er(OH)_*x*_. For the Er_2_TiO_5_ film, the intensity of Er 4*d*_5/2_ peak corresponding to Er_2_TiO_5_ was larger than that of Er_2_O_3_. Furthermore, the Er 4*d*_5/2_ peak corresponding to Er_2_O_3_ for Er_2_TiO_5_ sample had a lower intensity compared with Er_2_O_3_ sample. These results are due to the reaction of TiO_*x*_ with the Er atom to form an Er_2_TiO_5_ structure. The O 1*s* spectra of the Er_2_O_3_ and Er_2_TiO_5_ films are shown in Figure 2b with their appropriate peak curve-fitting lines. The O 1*s* signal comprised three peaks at 530.2, 531, and 532.7 eV, which we assign to Er_2_O_3_[14], Er_2_OTi_5_, and Er(OH)_*x*_, respectively. The intensity of O 1*s* peak corresponding to Er(OH)_*x*_ bonding for the Er_2_O_3_ film was larger in comparison with the Er_2_TiO_5_ film, indicating that the reaction between the Er and water caused hydroxide units in the film. The O 1*s* peak of the Er_2_TiO_5_ film exhibits a large intensity peak corresponding to Er_2_TiO_5_ and two small intensity peaks corresponding to Er_2_O_3_ and Er(OH)_*x*_. This result indicates that the reaction of TiO_*x*_ with Er atom forming an Er_2_TiO_5_ film suppresses the formation of Er(OH)_*x*_.

**Figure 3:**
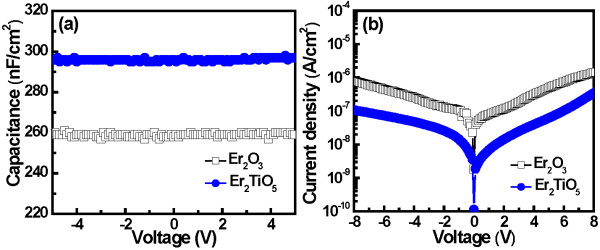
**Capacitance-voltage curves (a) and current–voltage characteristics (b) of Al/Er**_**2**_**O**_**3**_**/TaN and Al/Er**_**2**_**TiO**_**5**_**/TaN structure devices.**

Figure 3a shows the *C*-*V* curves of the Al/Er_2_O_3_/TaN and Al/Er_2_TiO_5_/TaN capacitor devices. The Al/Er_2_TiO_5_/TaN capacitor exhibited a higher capacitance density than the Al/Er_2_O_3_/TaN one. In addition, the *κ* value of the Er_2_O_3_ and Er_2_TiO_5_ dielectric films is determined to be 13.7 and 15.1, respectively. Figure 3b depicts the current–voltage characteristics of the Al/Er_2_O_3_/TaN and Al/Er_2_TiO_5_/TaN devices. The Al/Er_2_TiO_5_/TaN device exhibited a lower leakage current than the Al/Er_2_O_3_/TaN device. This result is attributed to the formation of a smooth surface at the oxide/channel interface.

**Figure 4:**
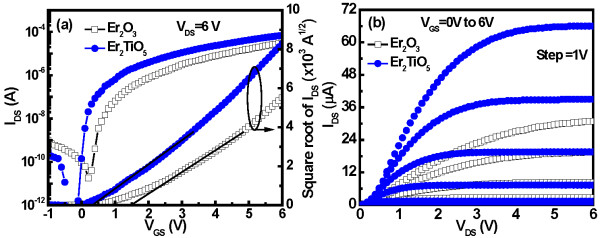
**Transfer and output characteristics.** Transfer characteristics (*I*_DS_-*V*_GS_) (**a**) and output characteristics (*I*_DS_-*V*_DS_) (**b**) of high-*κ* Er_2_O_3_ and Er_2_TiO_5_ a-IGZO TFT devices.

The transfer characteristics of the a-IGZO TFT devices using Er_2_O_3_ and Er_2_TiO_5_ gate dielectrics were shown in Figure 4a. The *V*_TH_ value of the Er_2_O_3_ and Er_2_TiO_5_ a-IGZO TFT devices is 1.5 and 0.39 V, whereas the *I*_on_/*I*_off_ ratio is 1.72 × 10^6^ and 4.23 × 10^7^, respectively. The moisture absorption of the Er_2_O_3_ film generates a rough surface due to the formation of Er(OH)_*x*_, thus causing degradation in the electrical characteristics. Furthermore, the *I*_off_ current can be improved by bottom gate pattern to reduce the leakage path from the gate to the source and drain. Furthermore, the *μ*_FE_ of the Er_2_O_3_ and Er_2_TiO_5_ TFT devices is 6.7 and 8.8 cm^2^/Vs. This result is due to the smooth roughness at the oxide-channel interface [15]. The subthreshold swing (SS) of the Er_2_O_3_ and Er_2_TiO_5_ TFT devices is 315 and 143 mV/dec, respectively. The titanium atoms can effectively passivate the oxygen vacancies in the Er_2_TiO_5_. The effective interface trap state densities (*N*_it_) near/at the interface between the dielectric and IGZO were estimated from the SS values. By neglecting the depletion capacitance in the active layer, the *N*_it_ can be calculated from the relationship [6]:

(1) $$N_{\mathrm{it}}=\left(\frac{\mathrm{SS}}{\ln10}\frac{q}{\mathrm{kT}}-1\right)\frac{C_{\mathrm{ox}}}{q},$$

where *q* is the electronic charge; *k*, the Boltzmann's constant; *T*, the temperature; and *C*_ox_, the gate capacitance density. The *N*_it_ values of IGZO TFTs using Er_2_O_3_ and Er_2_TiO_5_ gate dielectrics are about 6.92 × 10^12^ and 2.58 × 10^12^ cm^−2^, respectively. Figure 4b shows the output characteristics of the a-IGZO TFT devices using the Er_2_O_3_ and Er_2_TiO_5_ gate dielectrics. As is seen, the driving current increases significantly for the Er_2_TiO_5_ dielectric material. This outcome may be attributed to the higher mobility and smaller threshold voltage.

**Figure 5:**
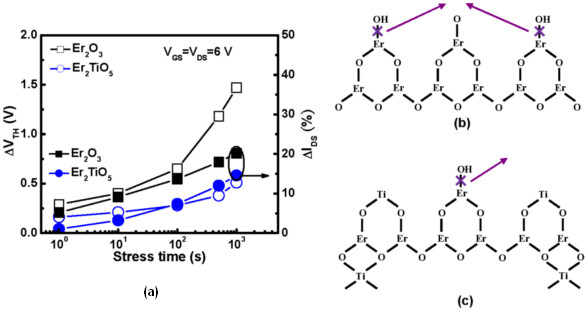
**Threshold voltage and drive current degradation and structural model.** (**a**) Threshold voltage shift and current drive degradation as a function of stress time for high-*κ* Er_2_O_3_ and Er_2_TiO_5_ a-IGZO TFT devices. Structural model of the (**b**) Er_2_O_3_ surface and (**c**) Er_2_TiO_5_ surface.

To explore the reliability of an a-IGZO transistor, the dc voltage was applied to the high-*κ* Er_2_O_3_ and Er_2_TiO_5_ a-IGZO TFT devices. Figure 5a shows the threshold voltage and drive current degradation as a function of stress time. The voltage stress was performed at *V*_GS_ = 6 V and *V*_DS_ = 6 V for 1,000 s. The shift in threshold voltage and the degradation in drive current are associated with the trap states in the dielectric layer and the interface between the dielectric film and channel layer [16]. The large *V*_TH_ shift (1.47 V) of the Er_2_O_3_ TFT can be due to more electrons trapping near/at the interface between the Er_2_O_3_ and IGZO layer [6], whereas the low *V*_TH_ shift (0.51 V) of the Er_2_TiO_5_ TFT device may be attributed to the reduction of the trapped charge in the film. With increasing *V*_GS_, interface states are substantially generated, which are normally regarded to be Er dangling bonds (=Er•), originating from the dissociation of weak Er-OH bonds at the oxide/channel interface. The dissociation of Er-OH bonds under dc stressing is proposed to be associated by the electrons in the oxide surface as follows:

(2) $$\begin{array}{l} =\text{Er}-\mathrm{OH}\;+e^{-}\to =\text{Er•}+{\;\mathrm{OH}}^{-}{2\mathrm{OH}}^{-}\to {\;H}_{2}O\;+\;O\;+\;2e^{-} \end{array}$$

The physical model to be presented is based on the structure of the Er_2_O_3_ and Er_2_TiO_5_ surfaces, as schematically depicted in Figure 5b,c, respectively. Briefly speaking, during dc stress, hydroxyl ions (OH^–^) are released from the erbium hydroxide (Er-OH) by breaking the Er-OH bonds. The electrons in the oxide have gained enough energy from the applied gate and drain voltages. They collide with strained Er-O-Er or Er-O-Ti bonds to generate trapped charges in bulk oxide, causing a threshold voltage shift. On the other hand, a-IGZO TFT with the Er_2_O_3_ dielectric has a larger drive current degradation than that with the Er_2_TiO_5_ one. The hygroscopic nature of RE oxide films forming hydroxide produces oxygen vacancies in the gate dielectric, leading to a larger flat-band voltage shift and higher leakage current [11]. The incorporation of Ti into the Er_2_O_3_ dielectric film can effectively reduce the oxygen vacancies in the film.

## Conclusions

In conclusion, we have fabricated a-IGZO TFT devices using the Er_2_O_3_ and Er_2_TiO_5_ films as a gate dielectric. The a-IGZO TFT incorporating a high-*κ* Er_2_TiO_5_ dielectric exhibited a lower *V*_TH_ of 0.39 V, a larger *μ*_FE_ of 8.8 cm^2^/Vs, a higher *I*_on_/*I*_off_ ratio of 4.23 × 10^7^, and a smaller subthreshold swing of 143 mV/dec than that of Er_2_O_3_ dielectric. These results are attributed to the addition of Ti into the Er_2_O_3_ film passivating the oxygen vacancies in the film and forming a smooth surface. Furthermore, the use of Er_2_TiO_5_ dielectric film could improve the stressing reliability. The Er_2_TiO_5_ thin film is a promising gate dielectric material for the fabrication of a-IGZO TFTs.

## Competing interests

The authors declare that they have no competing interests.

## Authors’ contributions

FHC designed the experiment, measured the a-IGZO TFT device data, and drafted the manuscript. JLH provided useful suggestions and helped analyze the characterization results. YHS performed the experiment and measured the electrical characteristics. YHM helped in the technical support for the experiments. TMP supervised the work and finalized the manuscript. All authors read and approved the final manuscript.
